# Carbonaceous aerosol tracers in ice-cores record multi-decadal climate oscillations

**DOI:** 10.1038/srep14450

**Published:** 2015-09-28

**Authors:** Osamu Seki, Kimitaka Kawamura, James A. P. Bendle, Yusuke Izawa, Ikuko Suzuki, Takayuki Shiraiwa, Yoshiyuki Fujii

**Affiliations:** 1 Institute of Low Temperature Science, Hokkaido University, Hokkaido, Japan; 2 School of Geography, Earth and Environmental Sciences, University of Birmingham, UK; 3 Faculty of Science, Tokyo Metropolitan University, Tokyo, Japan; 4 National Institute of Polar Research, Tokyo, Japan

## Abstract

Carbonaceous aerosols influence the climate via direct and indirect effects on radiative balance. However, the factors controlling the emissions, transport and role of carbonaceous aerosols in the climate system are highly uncertain. Here we investigate organic tracers in ice cores from Greenland and Kamchatka and find that, throughout the period covered by the records (1550 to 2000 CE), the concentrations and composition of biomass burning-, soil bacterial- and plant wax- tracers correspond to Arctic and regional temperatures as well as the warm season Arctic Oscillation (AO) over multi-decadal time-scales. Specifically, order of magnitude decreases (increases) in abundances of ice-core organic tracers, likely representing significant decreases (increases) in the atmospheric loading of carbonaceous aerosols, occur during colder (warmer) phases in the high latitudinal Northern Hemisphere. This raises questions about causality and possible carbonaceous aerosol feedback mechanisms. Our work opens new avenues for ice core research. Translating concentrations of organic tracers (μg/kg-ice or TOC) from ice-cores, into estimates of the atmospheric loading of carbonaceous aerosols (μg/m^3^) combined with new model constraints on the strength and sign of climate forcing by carbonaceous aerosols should be a priority for future research.

A better understanding of the forcing and feedback mechanisms that drive decadal to centennial climate variability is vital to improve the accuracy of near future climate projections by model simulations. External forcings by solar irradiance and volcanic aerosols and internal oscillations in the Earth’s climate system account for a significant degree of the natural (non-anthropogenic) component of temperature anomalies over such timescales^1^,^2^,^3^ (Fig. 1a,b). However, model simulations, which attempt to incorporate the known principal forcings (solar, volcanic, inorganic aerosols and greenhouse gases), tend to underestimate the amplitude of historical temperature fluctuations over the past 1000 years^4^. In particular, the gap between real world climate changes in the past and model estimates is profound in the Arctic^5^.

Better constraints on historical changes in carbonaceous aerosols, which consist of light absorbing and light-scattering components^6^, may help to reconcile such proxy-model amplitude mismatches since, along with clouds, their microphysical processes and transport mechanisms constitute the greatest uncertainty in both models and observations^7^. Carbonaceous aerosols (including light absorbing black carbon (BC) and brown carbon (BrC)) are typically produced as pyrolysis products of fossil fuel combustion and biomass burning processes^8^,^9^,^10^. BrC is also emitted to the atmosphere by the deflation of soil organic matter, which includes light absorbing humic substances. Carbonaceous aerosols have long been ascribed a negligible or slightly negative forcing over biomass burning regions in climate models, with organic matter treated as light-scattering (and the light-absorbing BrC component overlooked)^11^. However, recent observational and smog-chamber studies, indicate that the net direct radiative forcing of carbonaceous aerosols (including the BC, BrC and light-scattering organic matter components), can be significantly positive over both anthropogenic source and biomass burning regions^12^,^13^. A recent model shows that, overall, carbonaceous aerosols may currently be yielding a global net warming effect (+0.65 W/m^2^), comparable to that of methane^12^.

A recent compilation of charcoal data sets from lake and peat sediment as well as ice core archives shows a link between wildfires and climate over centennial time scales^14^,^15^. However, such studies are challenged by sparse data coverage in high-latitudes and Asia and do not capture the non-biomass burning component (e.g. soil organic matter) of BrC deflated to the atmosphere during dust storms. Thus the hemispheric scale role of carbonaceous aerosols in decadal scale climate change is still highly uncertain. Here we provide two Northern Hemisphere (NH) ice-core records containing biomass burning-, soil bacterial- and plant wax-tracers and compare these with paleoclimate data to investigate the relationship of these carbonaceous aerosols to climate since 1550 AD.

We utilize newly generated (dicarboxylic acid in Site-J and monocarboxylic acid in Ushkovsky) and previously published organic tracer records^16^,^17^ derived from Greenland Site-J (66°51.9′N, 46°5.9′W, altitude: 2030 m) and Kamchatka Ushkovsky (56.04°N, 160.28°W, altitude: 3903 m) (Fig. 2) to investigate the natural variability of carbonaceous aerosols in the northern high latitudes and to highlight empirical links to climate over decadal time scales.

## Results

### Organic tracers of carbonaceous aerosols

Concentrations of specific organic tracers: soil bacteria derived long-chain (C_26_–C_30_) dicarboxylic acids (hereafter di-acids), higher plant leaf-wax derived long-chain (C_26_–C_30_) monocarboxylic acids (hereafter leaf-waxes) and biomass burning derived levoglucosan are applied to reconstruct changes in the deflation and transport of soil organic matter (di-acids and leaf-waxes) and biomass burning particles (levoglucosan and leaf-waxes).

Long-chain di-acids are produced in soils by bacterial oxidation of plant-derived fatty acids^18^. These compounds are ubiquitous, but often overlooked, in aerosols and have been proposed as a specific molecular tracer for aeolian transport of soil organic matter^18^,^19^,^20^. A weekly sampling campaign, for aerosol analyses, was conducted in Spring 1991 at the Alert station (82°30′5′′N, 62°20′20′′W), Arctic^19^ (Fig. S1). The Alert data provide evidence that, in addition to established deposition of mineral dusts, soil organic matter baring dust is also long-range transported to the Arctic from lower latitudes, during the spring dust storm season in Asia.

Levoglucosan is a major pyrolysis (>250 °C) product of the cellulose and hemicellulose that typically comprises 50–70% of dry wood^21^ and is emitted to the atmosphere by the combustion of woody and grassy components of biomass^22^,^23^,^24^. Levoglucosan is ubiquitous in the atmosphere from the Arctic to Antarctic^25^,^26^. Correlations between levoglucosan and black carbon concentrations are observed in the NEEM ice core from Greenland during the preindustrial era and in modern aerosols collected from the European Arctic (Zeppelin atmospheric observatory)^27^. Previous studies have demonstrated that levoglucosan preserved in ice cores from Greenland and Kamchatka could provide historical records of biomass burning and boreal forest fires^15^,^17^,^28^. However, we note that in the NEEM ice core the levoglucosan record does not correlate with that of black carbon during the past 200 years when fossil fuel combustion becomes a major source of black carbon^15^. Overall these results suggest that levoglucosan is a good indicator of biomass burning derived black carbon as long as the signal is not overprinted by fossil fuel combustion.

Long-chain (C_26_–C_30_) mono-carboxylic acids are major components of plant leaf waxes and are ubiquitous in the global atmosphere^20^,^29^,^30^, including in the Arctic region^25^. Leaf-waxes are also amongst the major molecular components directly emitted with biomass burning particles^31^,^32^. A three-year observation of aerosols in the North Atlantic region suggests that biomass burning is a major source of leaf-waxes transported to remote sites^29^. Additionally, these compounds are ubiquitous in soils and high atmospheric concentrations of leaf-waxes have been reported at a remote sampling station (Chichi-Jima Island) in the western North Pacific, during spring Asian dust plume events^20^. Thus, leaf-waxes are long-range transported to remote sites and can be used as tracers for both biomass burning particulates and soil organic matter inputs to the atmosphere^20^,^29^.

### Factors controlling the concentration of organic tracers in ice cores

**Figure 1:**
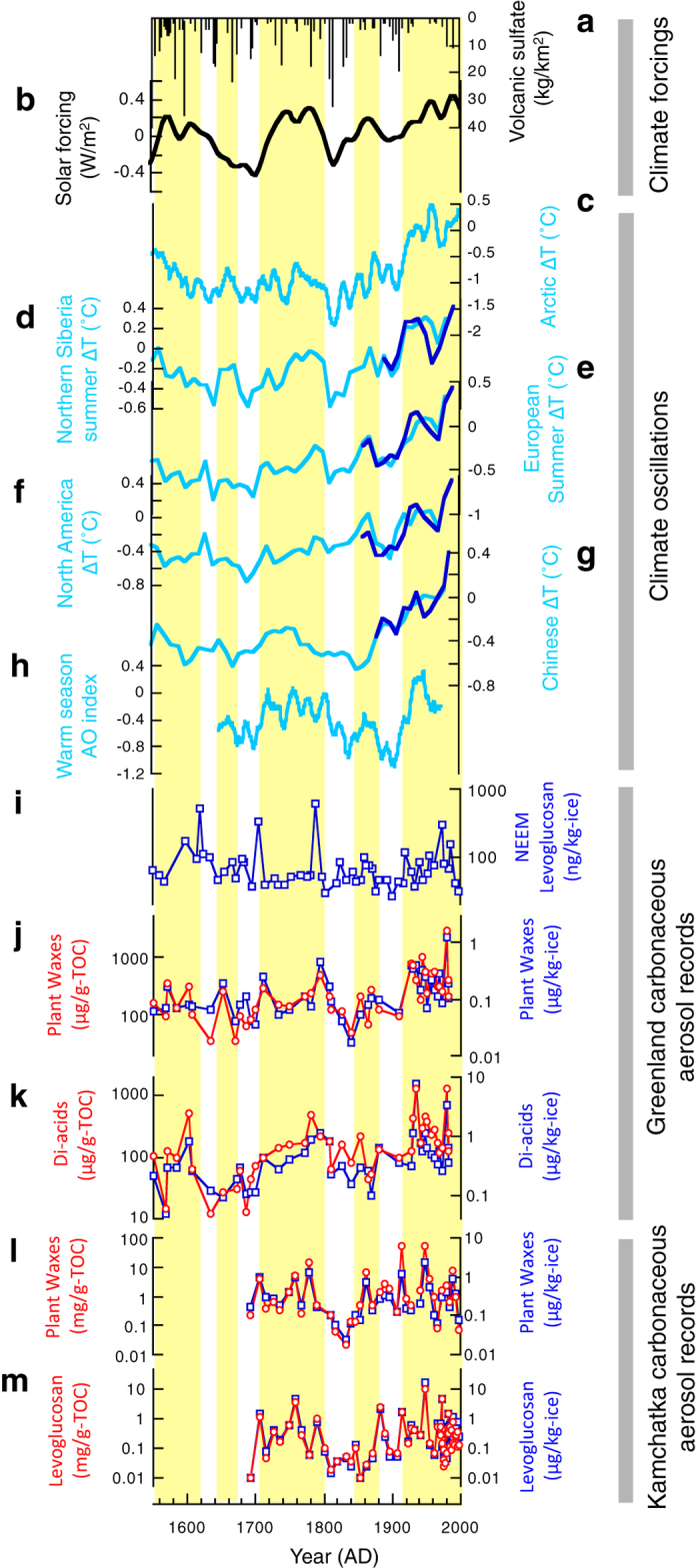
Comparison of carbonaceous aerosol tracers, from the Greenland Site-J and Kamchatka Ushkovsky ice cores, and climate reconstructions over the past 450 years. (**a**) Volcanic radiative forcing^2^. (**b**) Solar radiative forcing^1^. (**c**) Reconstructed Arctic temperatures^39^. (**d**) Reconstructed Northern Siberian summer temperatures^40^. (**e**) Reconstructed European summer temperatures^40^. (**f**) Reconstructed North American annual mean temperatures^40^. (**g**) Reconstructed Chinese summer temperatures^40^. The blue lines in figs d–g represent the instrumental temperature record. (**h**) Reconstructed warm season Arctic Oscillation (AO) index^47^. (**i**) Concentration of long-chain monocarboxylic acids (leaf-waxes) in the Site-J ice core^16^. (**i**) Concentrations of levoglucosan in NEEM ice core^15^. (**j**) Concentration of long-chain monocarboxylic acids (leaf-waxes) in the Site-J ice core^16^. (**k**) Concentrations of long-chain dicarboxylic acids (di-acids) in the Site-J ice core. (**l**) Concentrations of long-chain monocarboxylic acids (leaf-waxes) in the Ushkovsky ice core. m) Concentrations of levoglucosan in Ushkovsky ice core^13^. Blue square and red circle in figs i-l show concentrations per kg-ice and TOC-normalized concentrations of organic tracers, respectively. Yellow shading represents relatively warm periods.

Concentrations of organic tracers change by over three orders of magnitude in the Greenland Site-J and Kamchatka Ushkovsky ice cores between 1550 and 2000 AD (Fig. 1j–m). Organic tracers extracted from an ice core reflect the chemical composition of materials that are deflated to the atmosphere on regional scales, transported and mixed by aeolian processes, scavenged from the air column by wet (snow) and dry deposition, and incorporated into firn and then glacial archives. Thus, the concentrations of our ice core organic tracers are a function of variations in external inputs (controlled by atmospheric loading and transport pathways) of carbonaceous aerosols and potentially *in situ* glacial processes that will also affect the total organic carbon (TOC) composition of the samples, e.g., accumulation rate, summer melt events, microbial activity, etc. Therefore, we deconvolve the signal of external inputs of carbonaceous aerosols from overprinting by *in situ* glacial processes (Fig. 1j–m) by normalizing concentrations to TOC. It should be noted that accumulation rates in the ice cores do not significantly change over the studied periods compared to the concentrations of organic tracers^33^,^34^,^35^. Moreover, we note that our TOC and all the organic tracer records do not significantly correlate with measured melt events^36^,^37^ (Table S1), suggesting that any possible effects of accumulation rate on concentrations of organic tracers is negligible. Thus, we conclude that the concentrations of our organic tracers are not significantly biased by accumulation rates and/or summer melt events. We also note that the organic tracers we utilize cannot be generated *in-situ* in snow (e.g. by bacterial activity).

**Figure 2:**
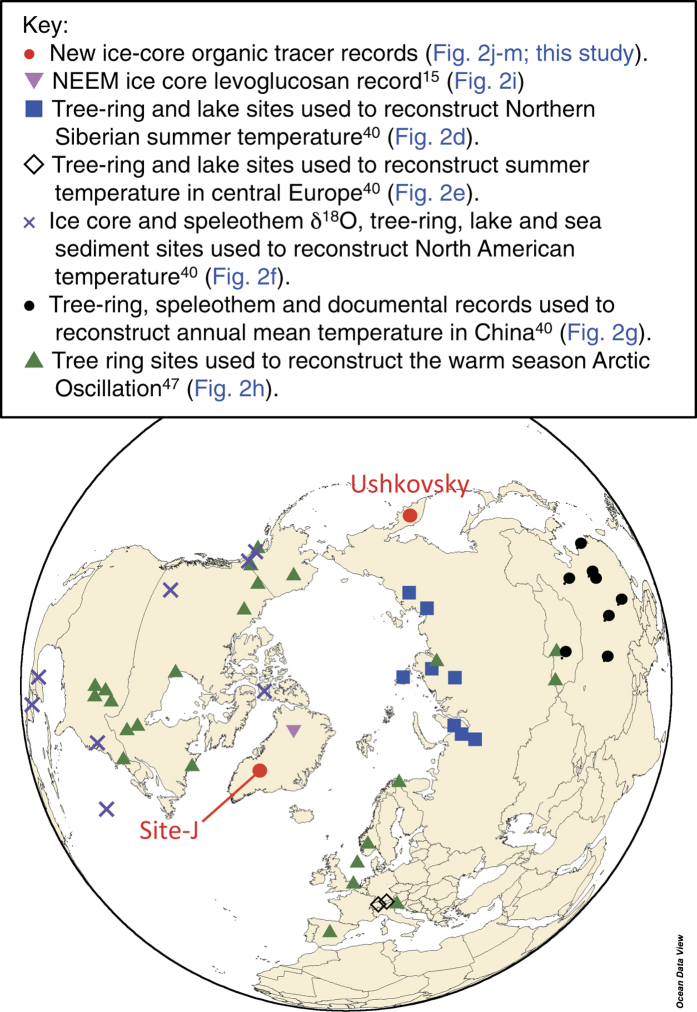
Map of the Northern Hemisphere showing locations of the ice core samples used in this study. Fig. 1 was made with Ocean Data View software^62^. Sites used for reconstruction of Arctic temperatures are shown in Fig. S5 (ref. 39).

We find that all the organic tracer records show similar variations in temporal patterns with fluctuations of several orders since 1550 CE. Especially, a pronounced decreases in concentrations are observed in the coldest periods of the little ice-age in the early 17^th^ century (in Greenland) and in the early 19^th^ century (in Greenland and Kamchatka). Peak concentrations in organic tracers are observed in both ice cores during 1920–1960′s when prominent warming occurred in the Arctic region. The coherence of the ice core records in the period of mutual overlap from 1690′s onwards is remarkable (especially the decrease in the early 19^th^ century) considering both the different sources of organic tracers and geographical separation (>6000 km) between the Greenland and Kamchatka sites (Fig. 2). In the Greenland record, prominent peaks in leaf wax concentrations (e.g., ca. 1700, 1800 and 1080 AD) are also recognized in the levoglucosan record in the NEEM ice core (Fig. 1i), northern Greenland^14^.

This observed coherence between organic tracers and climate records holds true, even after normalization to TOC to minimize the effect of any *in-situ* ice core processes. In order to quantify the effects of carbonaceous aerosols loading on regional climate since 1550 CE it would be necessary to translate concentrations of organic tracers (in units of μg/kg-ice or TOC) from ice-cores, into estimates of the atmospheric loading of carbonaceous aerosols (in units of μg/m^3^). Unfortunately, a lack of empirical data (e.g. the proportion of the organic tracers to the total carbonaceous aerosol inventory; conversion of concentrations from ice to firm and snow; dry and wet deposition efficiencies) means that this process currently contains too many fundamental uncertainties to be attempted. We note that organic tracers compromise a small portion of organic matter in ice cores (typically < 1%). Despite this, due to their source specificity and recalcitrance, these tracers are an established and powerful tool for tracing inputs of terrestrial material and have been extensively applied to sediments and aerosols^20^,^29^,^38^. Overall, the most parsimonious explanation for our measured order of magnitude changes in ice core concentrations of organic tracers (Fig. 1j–m) is that they do record a signal of large-scale changes in the atmospheric loading of carbonaceous aerosols in the high latitudinal Northern Hemisphere.

### Comparison of ice core records and climate reconstructions

To examine the extent to which ice core records of organic tracers link to climate, we compared our records with the best available climate reconstructions from the different parts of the mid- to high-latitudinal Northern Hemisphere, including mean annual temperatures from the Arctic^39^, North America^40^ and China^40^ and warm season temperatures from Europe^40^ and N. Siberia^40^ (Fig. 1c–1h and Table 1). The assignation of mean annual vs warm season temperatures depends on the available proxy records and the assessment of seasonal bias by Ljungqvist (2010) (ref. 40). Most high latitude data, except for the Greenland borehole and δ^18^O ice-core records, have a clear bias towards warm season temperatures^40^. Mean annual mean temperatures in the mid-latitudes may also contain an inherent bias towards the warm season, since paleoclimate data that are calibrated to reconstruct regional mean annual temperatures are often derived from a proxy substrate which was produced primarily during a summer growth season^40^. For instance, 8 records were used to reconstruct annual mean temperature compilation for North America, but 3 of the 8 records are explicitly summer temperature reconstructions^40^.

Our ice core records (Fig. 1j–m) significantly correlate with historical temperatures in proximal regions; for example, the Greenland leaf wax and di-acid data have significant correlations with summer temperatures in Central Europe (Fig. 1e) (R = 0.60 to 0.59, *p *< 0.01) and annual mean temperatures in North America^40^ (R = 0.50 to 0.49) (Fig. 1f) while the Kamchatka leaf wax and levoglucosan records correlate with summer temperatures in northern Siberia (Fig. 1d) (R = 0.53 to 0.39, *p *< 0.01) and mean annual temperatures in China^40^ (Fig. 1g) (R = 0.50 to 0.39, *p *< 0.01) (Table 1). Further evidence of a Northern Hemisphere link between regional climate and biogenic aerosol loading comes from records of ammonium and formate in a glacier ice core record from southern Russia^41^. The relatively high correlations of all the ice core organic tracers (Fig. 1j–m) with Arctic annual mean temperatures^39^ (Fig. 1c) (R = 0.52 to 0.35, *p *< 0.05) and summer temperatures in Northern Siberia^40^ (Fig. 1d) (R = 0.59 to 0.39, *p *< 0.01) suggests a strong empirical relationship between the ice core records and high latitude climate. This is consistent with modern observations; that is frequencies of boreal forest biomass burning and soil dust plume events increase during the warm season (spring to summer) when the arid regions of China and the Eurasian boreal forests are thought to act as major source regions of soil dust and biomass burning tracers preserved in Greenland and Kamchatka ice cores^15^,^17^,^42^.

## Discussion

The outstanding feature of our organic tracer records are the orders of magnitude increases in concentrations coeval with warmer periods in observed in Northern hemisphere temperature records (Fig. 1). This observation contrasts with the Greenland ice core records of the major inorganic aerosols (e.g. volcanic sulfate), which increase during cold climatic phases^2^,^43^. One possible mechanism that could affect hemispheric scale control on emissions, loadings and long-range transport of carbonaceous aerosols in high latitude regions, is the Arctic Oscillation (AO), a key feature of climate variability in the Northern Hemisphere. AO significantly influences regional and hemispheric wind regimes and climate^44^,^45^,^46^, especially in the mid and high latitudes. Supporting this hypothesis, regional temperature records^39^,^40^, which correlate with our ice core records, also show significant correlations with reconstructed warm season AO (ref. 47) (R = 0.41 to 0.34, Fig. 1h and Table 1) while we did not find significant correlations with the reconstructed winter AO (ref. 48) and other decadal climate oscillations such as the Atlantic Multidecadal Oscillation^49^ or Pacific Decadal Oscillation^50^. Further support for this hypothesis comes from significant correlations between all the ice core organic tracers, and summer temperatures in northern Siberia^40^ (R = 0.59 to 0.39, *p *< 0.01) where summer temperature is especially sensitive to summer AO (ref. 44) and thus could be a representative of warm season AO. However, the tree ring based warm season AO reconstruction^47^ does not significantly correlate with the Ushkovsky ice core records (R < 0.2, p > 0.05; Table 1). This weak correlation is possibly attributed to the fact that the Ushkovsky ice core records reflect aerosol inputs both from low latitudes (China) as well as high latitude areas (Siberia) as suggested by relatively high correlations of Ushkovsky data with the Chinese temperature record^40^ (Table 1). Alternatively, the low correlation may stem from the inherent uncertainty of the warm season AO reconstruction, which is reconstructed using tree ring data from different parts of the Northern Hemisphere, where sensitivity to summer AO is regionally variable.

The correlations of ice-core carbonaceous aerosol tracers with the AO and with climate in China are noteworthy given the important role of the Taklamakan desert as a noted source region for atmospheric dust^42^,^51^ (see Supplementary Information). Based on Sr and Nd isotopes, the Taklamakan desert in western China has been suggested as a major source of mineral dust deposited in high elevation sites of Greenland^42^. Taklamakan dust is uniquely lofted up to the upper troposphere, via strong regional katabatic winds and low pressure fronts, and can be transported more than one full circuit around the globe by the westerly jet^51^.

Overall, our organic ice core tracers record that over the last 450 years, warmer/positive AO phases resulted in increased deflation/long-range transport of biomass burning products and transport of soil organic matter (OM). There are two possible mechanisms by which AO could influence the deposition of carbonaceous aerosols over ice sheets in the high latitudinal Northern Hemisphere: 1) intensified transport from the source regions to ice core sites, and 2) enhanced emissions of carbonaceous aerosols in source regions during positive phases of AO. Support for enhanced transport, via intensified poleward winds, during the positive phase of AO comes from model simulations^52^.

Various lines of evidence support the 2^nd^ mechanism (enhanced emissions)^53^,^54^. A positive AO mode intensifies high pressure at mid-latitudes, inducing significant warming over northern Eurasia^44^, which leads to an increased biomass burning frequency in central Siberia^53^. Furthermore, based on backward air mass trajectory analyses, Kawamura *et al.* (2012) suggests that levoglucosan recorded in the Ushkovsky ice core mainly reflects historical changes in warm season biomass burning in Siberia, the Far East and northern China^17^. A wind erosion model^54^ suggests that emissions of dust in the Taklamakan desert, which is the major source of mineral dust deposited in high elevation sites of Greenland^42^, respond to changes in AO, i.e., a positive AO phase increases the dust storm frequency and intensity in the Taklamakan Desert. Also, our di-acid record shows coherence with dust data in the Dunde ice core, central China (Fig. S6), providing supporting evidence that Greenland ice cores, significantly reflect dust loading from the arid regions of China^55^.

**Figure 3:**
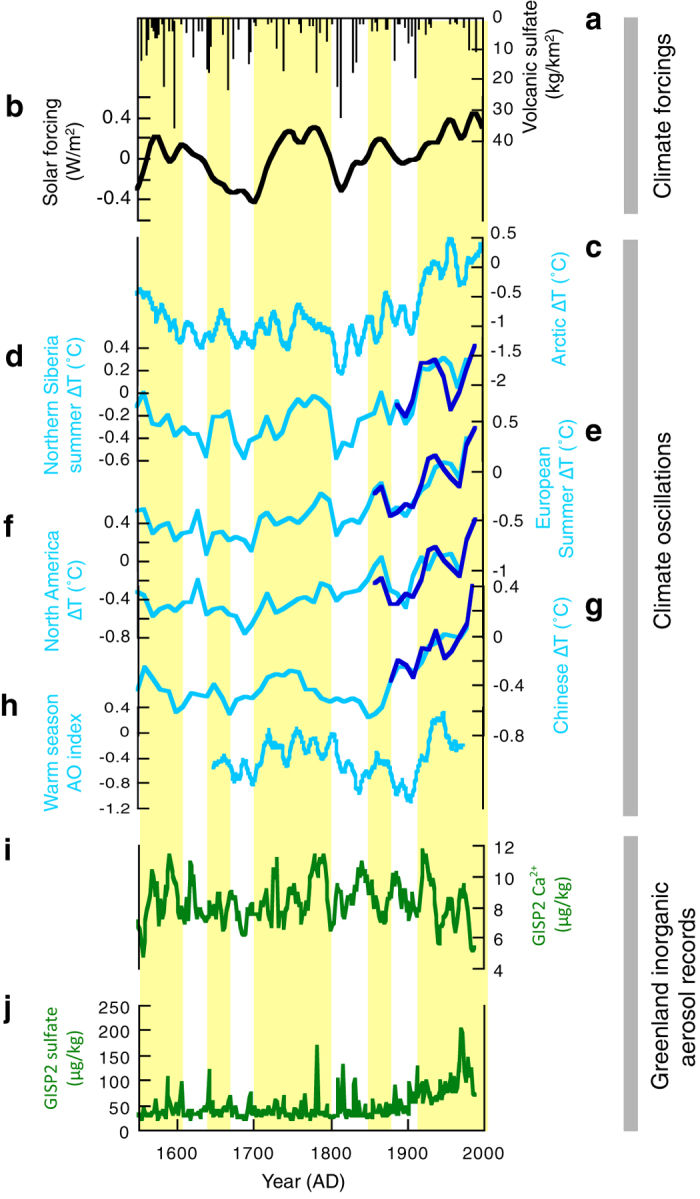
Comparison of inorganic aerosol tracers from the Greenland GISP2 and climate reconstructions over the past 450 years. (**a**) Volcanic radiative forcing^2^. (**b**) Solar radiative forcing^1^. (**c**) Reconstructed Arctic annual mean temperatures^39^. (**d**) Reconstructed Northern Siberian summer temperatures^40^. (**e**) Reconstructed European summer temperatures^40^. (**f**) Reconstructed North American annual mean temperatures^40^. (**g**) Reconstructed Chinese summer temperatures^40^. Blue lines in figs (**d–g**) represent instrumental temperature record. (**h**) Reconstructed warm season Arctic Oscillation (AO) index^47^. (**i**) Concentration of Ca^2+^ in the GISP2^43^. (**j**) Concentration of sulfate in GISP2^43^. Yellow shading represents relatively warm periods.

It is noteworthy that correlations between dust concentrations and warm season AO exists for the organic dust proxies but is insignificant for published Ca^2+^ mineral dust proxies^43^ (Fig. 3). This differential response is likely explained by a function of aerodynamic sorting of the <μm organics from the >μm mineral dusts^56^. In general, organic matter is enriched in the fine fraction dust particles whose diameters are submicron size, while diameters of mineral dust in the atmosphere have been reported to be several micrometers. Evidence to support such aerodynamic sorting is found in the spatial patterns of mineral and organic proxies in the Pacific Ocean surface sediments^56^. Because of a profuse supply of dust during the warm season^57^ to the upper-troposphere and subsequent long-range transport^51^, fine dust particles ultimately reach the high Arctic region.

In contrast, both models and observations suggest that most mineral dust from Asia is deposited underneath the path of the westerly jet and confined to the mid-latitudes while deposition of organic dust increases with latitude^51^,^56^. Thus, the organic-rich dust in the fine-fraction may be unique in encoding the signal of warm season AO in high-latitude ice cores, whilst such signals in mineral dust proxies are likely attenuated by greater rates of fall-out. We also note that recent agreement between models and observations suggest a potential forcing of AO by solar irradiance with negative/positive AO modes during solar minima/maxima^58^. In that context, it is noteworthy that our Greenland di-acid and plant wax records also correlate with solar irradiance^1^ (Table 1).

In summary our ice core organic tracer records likely reflect multi-decadal variations in the deflation of biomass burning products and soil organic matter and their long-range atmospheric transport from the key source regions. Moreover, northern Siberia^40^ and Arctic temperatures^39^ and the summer AO correlate with order of magnitude increases (decrease) in concentration of our organic tracers. In this context our data begs the question, do carbonaceous aerosols play a positive feedback role on multi-decadal time-scales via the AO?

Two possible positive amplifiers are: i) the direct effect of a net positive perturbation by atmospheric carbonaceous aerosols^12^ and ii) reduction of snow and ice surface albedo, directly through the deposition of light-absorbing aerosols^59^ and via carbonaceous aerosols acting as a substrate for *in-situ* microbial activity, which may further lower albedo^60^. However, fundamental uncertainties remain over the strength and sign of any carbonaceous aerosol forcing via the above mechanisms.

This work highlights the previously unexploited potential of terrestrial organic tracers in ice cores for palaeoclimatic applications and provides the first evidence of a link between carbonaceous aerosol loading in the atmosphere and multi-decadal climate changes. We hope this will open new avenues for ice core research and will prompt further proxy studies, including efforts to translate ice core concentrations of organic tracers to accurate estimates of atmospheric loading. Finally, new modeling constraints on the strength and sign of climate forcing by carbonaceous aerosols should be a priority for exploring possible feedbacks mechanisms and the implications for future climate change.

## Methods

### Ice cores chronologies

The chronology of the Site-J ice core was determined by summation of average annual accumulation rate obtained by volcanic signals. Full details are given in ref. 33. The chronology of the Ushkovsky ice core was determined by counting the annual layers of the seasonal oxygen isotopic signal (δ^18^O) for the upper part of the sequence (surface to 103.58 m in depth) whereas the chronology for deeper layers was determined by using a two-dimensional thermodynamic coupled model^34^,^35^.

### Organic tracer analyses

The Site-J and Ushkovsly ice core samples were cut every ~50 cm and were stored in a cold room (at −20 °C) until analysis. 1 cm surfaces of ice core sample were shaved off using a ceramic knife to avoid a possible contamination. Lipid compounds including leaf-waxes and di-acids were extracted with organic solvents from melted ice core samples. After acidifying the melted ice core sample (ca. 350 ml) with 6 M HCl, lipid compounds were extracted with methylene chloride/ethyl acetate (2:1) mixture. Extracts were saponified with a 0.5 M KOH/methanol. For the Ushkovsly ice core, total extracts were derivatized to TMS ethers or esters with bis(trimethylsulyl)trifuoroacetamide. Leaf waxes (long-chain monocarboxylic acids) in Ushkovsky ice core were measured by gas chromatograph/mass spectrometry (GC/MS). For the Site-J ice core, after the neutral fraction was removed from the extracts, the saponified fraction was acidified again with 6 M HCl. Di-acids were separated and derivatized to their corresponding methyl esters with 14% BF_3_ in methanol. The di-acid dimethyl esters in Site-J ice core were isolated by silica gel column chromatography and determined using a GC. Recovery of lipid compounds is 75–82%. Duplicate analyses of ice core samples showed that analytical error was within 15%.

### Time-Series and Correlations

The time-series data: biomarker data from the Site-J Greenland and Kamchatka ice cores; regional temperatures (Arctic, Northern Siberia, central Europe, North America and China)^39^,^40^, Summer AO^45^ and solar irradiance^1^ were available at different temporal resolutions. To facilitate correlations between time-series the data were re-sampled to common a time step, using a simple interpolation with a linear function in Analyseries 2.0 (ref. 61). No extrapolation was performed. The re-sampled time-series were visually inspected to ensure close resemblance of the re-sampled data to the original data and that spurious peak formation had not occurred. For the correlations between time-series a Spearman Rank Order correlation was performed on the overlapping period of the two data sets to be correlated. The correlation results and overlapping time periods are given in Table 1.

## Additional Information

**How to cite this article**: Seki, O. *et al.* Carbonaceous aerosol tracers in ice-cores record multi-decadal climate oscillations. *Sci. Rep.* **5**, 14450; doi: 10.1038/srep14450 (2015).

## Supplementary Material

Supplementary Information

## Figures and Tables

**Table 1:** Correlations of ice core aerosol records and climate reconstructions.

**1^st^ Variable**	**2^nd^ variable**	**Period**	**Correlation Coefficient**	**P Value**	**n**
**Arctic T**	**Leaf Waxes/TOC (Greenland)**	**1982 to 1550 CE**	**0.52**	**<0.01**	**55**
**Arctic T**	**Di-acids/TOC (Greenland)**	**1982 to 1550 CE**	**0.4**	**<0.01**	**55**
**Arctic T**	**Leaf Waxes/TOC (Kamchatka)**	**1997 to 1693 CE**	**0.35**	**<0.05**	**39**
**Arctic T**	**Levogl./TOC (Kamchatka)**	**1997 to 1500 CE**	**0.44**	**<0.01**	**39**
**Siberian T**_**summer**_	**Leaf Waxes/TOC (Greenland)**	**1982 to 1550 CE**	**0.59**	**<0.01**	**55**
**Siberian T**_**summer**_	**Di-acids/TOC (Greenland)**	**1982 to 1550 CE**	**0.5**	**<0.01**	**55**
**Siberian T**_**summer**_	**Leaf Waxes/TOC (Kamchatka)**	**1990 to 1693 CE**	**0.53**	**<0.01**	**38**
**Siberian T**_**summer**_	**Levogl./TOC (Kamchatka)**	**1990 to 1693 CE**	**0.39**	**<0.01**	**38**
**European T**_**summer**_	**Leaf Waxes/TOC (Greenland)**	**1982 to 1550 CE**	**0.6**	**<0.01**	**55**
**European T**_**summer**_	**Di-acids/TOC (Greenland)**	**1982 to 1550 CE**	**0.59**	**<0.01**	**55**
**European T**_**summer**_	**Leaf Waxes/TOC (Kamchatka)**	**1990 to 1693 CE**	**0.34**	**<0.05**	**38**
European T_summer_	Levogl./TOC (Kamchatka)	1990 to 1693 CE	0.24	<0.05	38
**North American T**	**Leaf Waxes/TOC (Greenland)**	**1982 to 1550 CE**	**0.5**	**<0.01**	**55**
**North American T**	**Di-acids/TOC (Greenland)**	**1982 to 1550 CE**	**0.49**	**<0.01**	**55**
North American T	Leaf Waxes/TOC (Kamchatka)	1990 to 1693 CE	–0.1	>0.05	38
North American T	Levogl./TOC (Kamchatka)	1990 to 1693 CE	0.04	>0.05	38
**Chinese T**	**Leaf Waxes/TOC (Greenland)**	**1982 to 1550 CE**	**0.33**	**<0.01**	**55**
**Chinese T**	**Di-acids/TOC (Greenland)**	**1982 to 1550 CE**	**0.33**	**<0.01**	**55**
**Chinese T**	**Leaf Waxes/TOC (Kamchatka)**	**1990 to 1693 CE**	**0.39**	**<0.01**	**38**
**Chinese T**	**Levogl./TOC (Kamchatka)**	**1990 to 1693 CE**	**0.5**	**<0.01**	**38**
**Summer AO**	**Leaf Waxes/TOC (Greenland)**	**1975 to 1650 CE**	**0.34**	**<0.05**	**42**
**Summer AO**	**Di-acids/TOC (Greenland)**	**1975 to 1650 CE**	**0.34**	**<0.05**	**42**
Summer AO	Leaf Waxes/TOC (Kamchatka)	1975 to 1693 CE	0.22	<0.05	**36**
Summer AO	Levogl./TOC (Kamchatka)	1975 to 1693 CE	0.18	>0.05	36
**Summer AO**	**Arctic T**	**1975 to 1650 CE**	**0.4**	**<0.01**	**42**
**Summer AO**	**Siberian T**_**summer**_	**1975 to 1650 CE**	**0.41**	**<0.01**	**42**
**Summer AO**	**European T**_**summer**_	**1975 to 1650 CE**	**0.34**	**<0.05**	**38**
**Summer AO**	**Chinese T**	**1975 to 1650 CE**	**0.35**	**<0.05**	**42**
Summer AO	North American T	1975 to 1650 CE	0.17	>0.05	42
**Solar irradiance**	**Leaf Waxes/TOC (Greenland)**	**1982 to 1550 CE**	**0.45**	**<0.01**	**55**
**Solar irradiance**	**Di-acids/TOC (Greenland)**	**1982 to 1550 CE**	**0.47**	**<0.01**	**55**
Solar irradiance	Leaf Waxes/TOC (Kamchatka)	1997 to 1693 CE	0.27	<0.05	39
Solar irradiance	Levogl./TOC (Kamchatka)	1997 to 1693 CE	0.16	>0.05	39

Correlation higher than 0.3 is shown in bold.
