# Supplementary material for: Carbonaceous aerosol tracers in ice-cores record multi-decadal climate oscillations
Source: Sci Rep. 2015 Sep 28;5:14450. doi: 10.1038/srep14450 (PMC4642758; doi:10.1038/srep14450)
Supplement: Supplementary Information [file srep14450-s1.pdf]

Supplemental information

Carbonaceous aerosol tracers in ice-cores record multi-decadal climate oscillations

Osamu Seki[1,*], Kimitaka Kawamura[1], James A.P. Bendle[2], Yusuke Izawa[1], Ikuko Suzuki[3], Takayuki Shiraiwa[1], Yoshiyuki Fujii[4]

[1] Institute of Low Temperature Science, Hokkaido University, Hokkaido, Japan.

[2] School of Geography, Earth and Environmental Sciences, University of Birmingham, UK.

[3] Faculty of Science, Tokyo Metropolitan University, Tokyo, Japan.

[4] National Institute of Polar Research, Tokyo, Japan.

*Corresponding author (seki@pop.lowtem.hokudai.ac.jp)

Table S1. Correlations of organic tracer concentrations with melt feature percentages in the Site-J and Ushkovsky ice cores.

1st Variable	*2nd variable*	*Period*	*Correlation Coefficient*	*P Value*	*n*
MWP (Site-J)	Leaf Waxes (Site-J)	1982 to 1550 CE	0.25	>0.05	43
MWP (Site-J)	Di-acids (Site-J)	1982 to 1550 CE	0.16	>0.05	43
MWP (Site-J)	TOC (Site-J)	1982 to 1550 CE	0.34	<0.05	43
MFP (Ushkovsky)	Leaf Waxes (Ushkovsky)	1997 to 1705 CE	0.13	>0.05	37
MFP (Ushkovsky)	Levogl. (Ushkovsky)	1997 to 1705 CE	0.15	>0.05	37
MFP (Ushkovsky)	TOC (Ushkovsky)	1997 to 1705 CE	0.03	>0.05	37

MWP = melt water percentage. MFP = melt feature percentage.

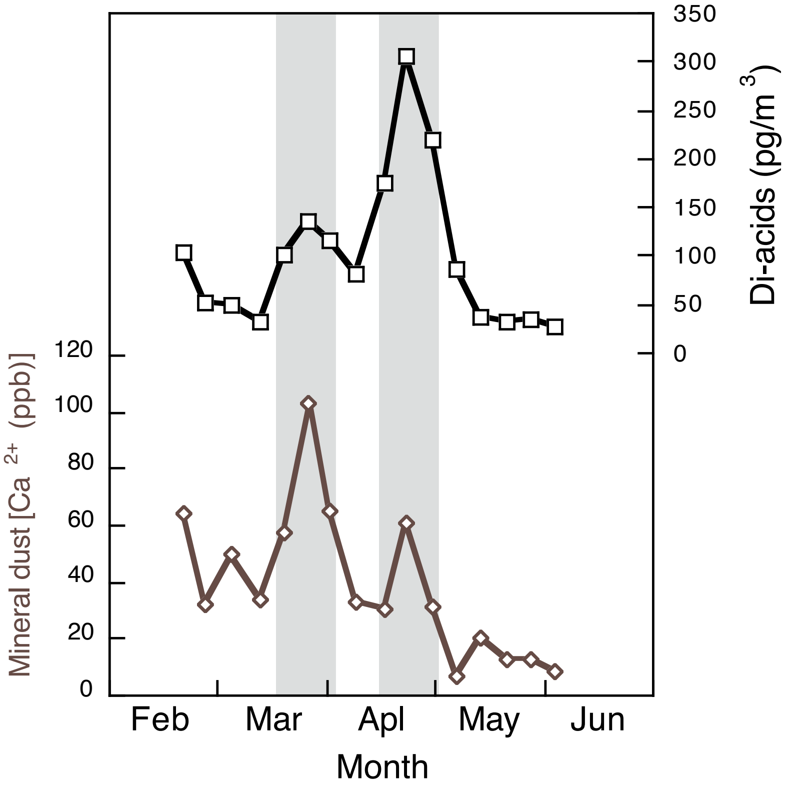

Figure S1. Seasonal changes in the concentrations of C_{23}-C_{26} di-carboxylic acids (di-acids) and mineral dust (Ca^{2+}) at Station Alert in 1991 (82°30’5’’N, 62°20’20’’W; Kawamura et al., 1996; Kawamura et al., 2010). Shaded areas represent dust plume events at Station Alert.

1. NASA NIMBUS-7 TOMS aerosol and Alert Station Back trajectory analyses.

To constrain the possible source areas for the peak organic dust inputs in observed in the Alert aerosol samples in 1991 (Fig. S1 above) we performed re-analyses of archived meteorological data using the NASA Giovanni visualization tool (developed and maintained by the NASA GES DISC) (Acker & Leptoukh, 2007) to map NASA TOMS 8 daily level 3 global 1.0°x1.25° gridded UV Aerosol Index data from the Nimbus-7 satellite. The results are given in Figures S2 and S3.

During the 1st dust storm event detected at the Alert station, from 6th March – 2nd April 1991, shows strong emissions from north Africa and Europe during the first three weeks (Fig. S2), with the Taklamakan desert becoming a clear hot-spot in the third week 20th-26th April, 1991) (Fig. S3). The relatively strong emissions from north Africa and lower emissions from Asia is consistent with the data at Alert, which shows relatively high mineral dust concentrations and low organic dust concentrations during the 1st dust storm event (Fig. S1). The 2nd plume event, from 9th to 30th April, 1991, builds from a relatively low dust period to peak emissions from 23rd-29th April 1991, with the Taklamakan desert and eastern China as clear hot-spots and with north Africa showing lower emissions (Fig. S3b). The relatively strong emissions from the Taklamakan and east Asia and lower emissions north Africa is again consistent with the data at Alert, which shows relatively high organic dust concentrations and low mineral dust concentrations measured at Alert during the 2nd 1991 Spring dust storm event.

Fig. S2: Dust storm event 1 (6th March – 2nd April, 1991).

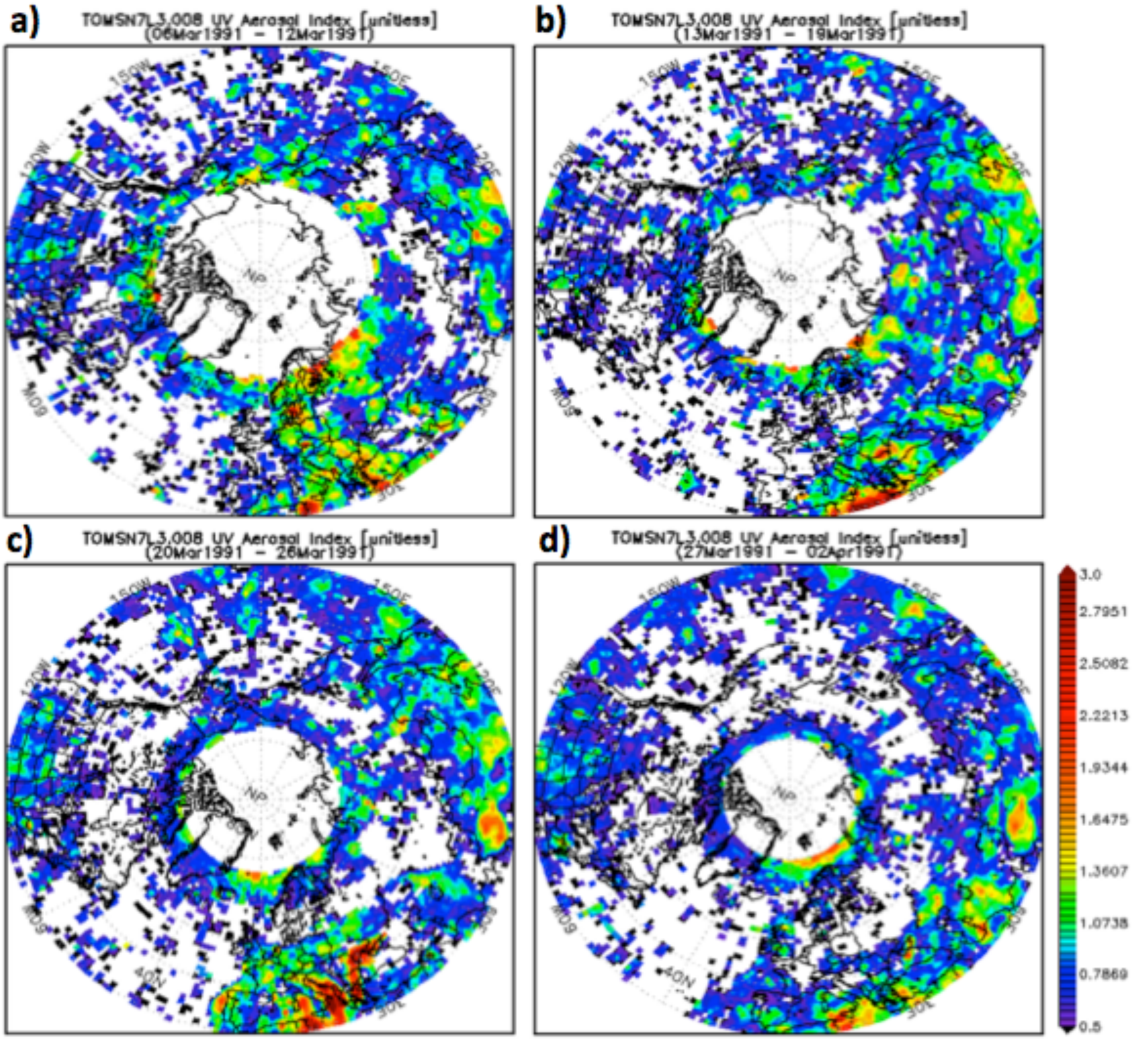

Fig. S3: Dust storm event 2 (9th April – 30th April, 1991).

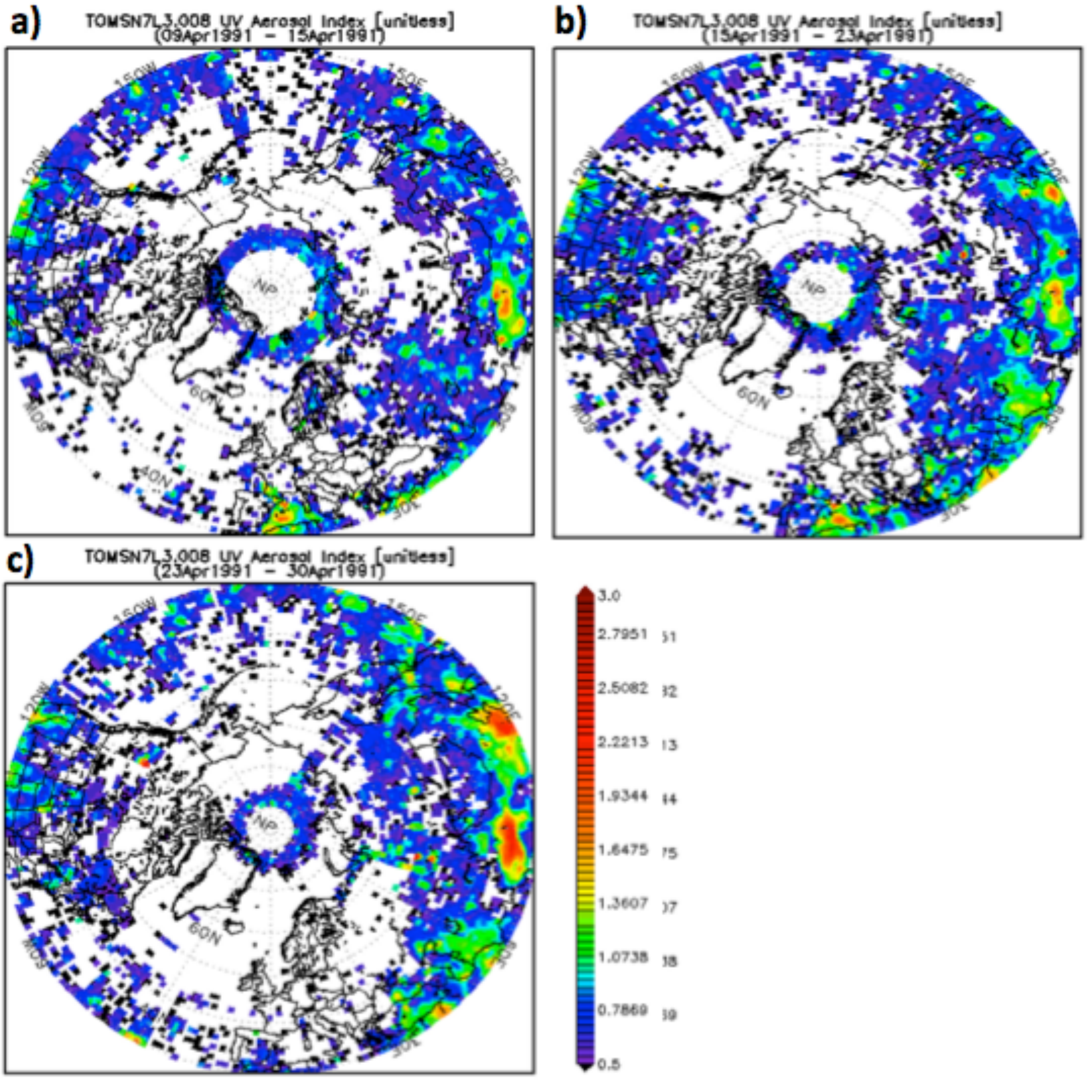

2. The Arctic Oscillation (AO) and the strength of the westerly jet.

The Arctic Oscillation (AO) is a prominent large-scale atmospheric circulation mode, expressed as the dominant empirical orthogonal function (EOF) of sea-level pressure (SLP) variability in the higher latitude of the Northern Hemisphere. The AO is a key feature of climate variability in the mid- and higher-latitudes of the Northern Hemisphere (NH), influencing climate in the entire region of the extratropics. The AO index is defined by surface atmospheric pressure pattern in mid to high latitude of the Northern Hemisphere; a positive (negative) AO index is characterized by low (high) surface pressure in the Arctic region with strong (weak) westerly jet above the western China and the Taklamakan desert (see Fig. S4).

Figure S4: *Response of zonal wind speeds (at 250mb) in the westerly jet stream above Asia during AO- and AO+ anomalies. Examples periods are1997-1998 (AO-) and 1989-1992 (AO+). Plots were made using NOAA reanalyses data.*

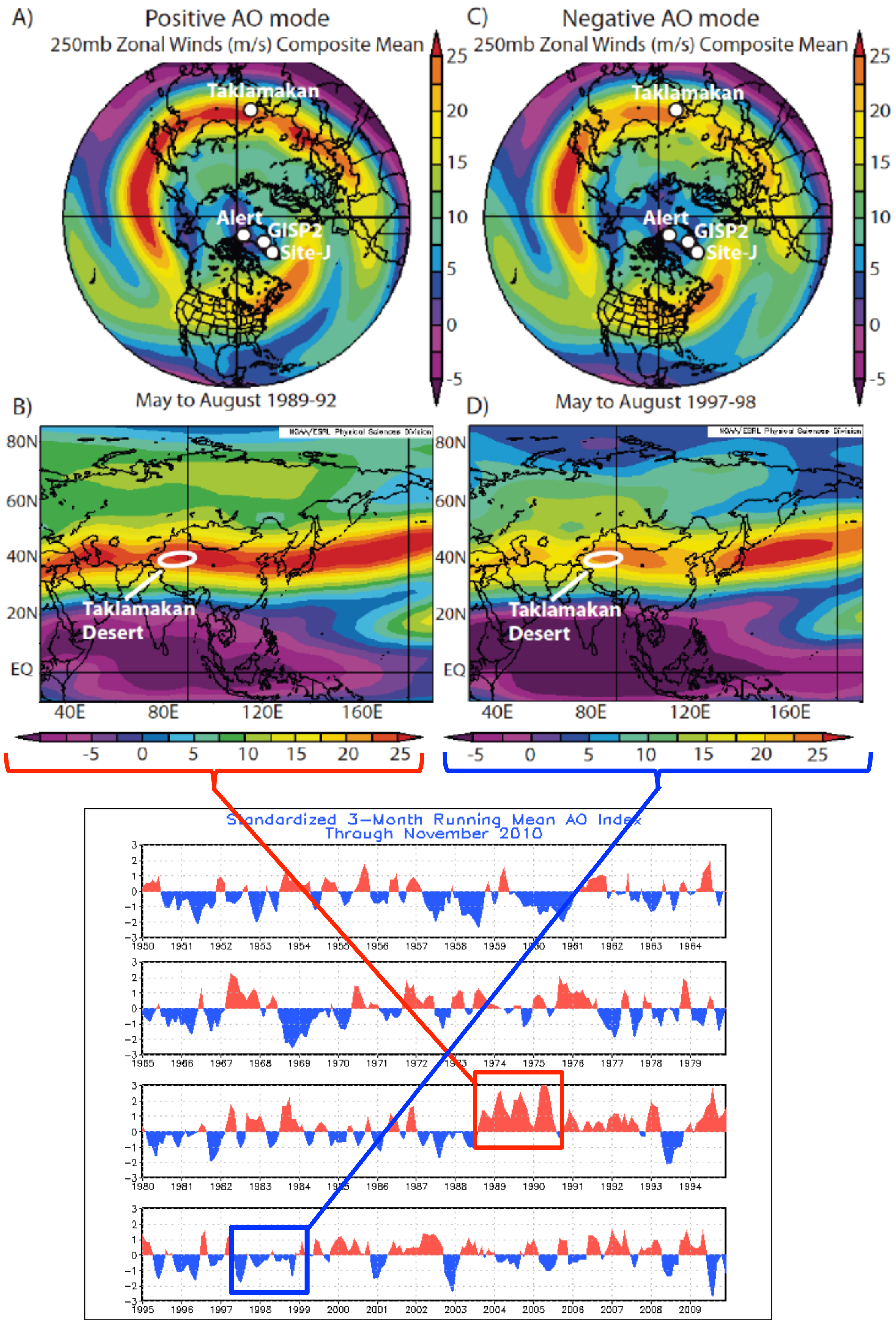

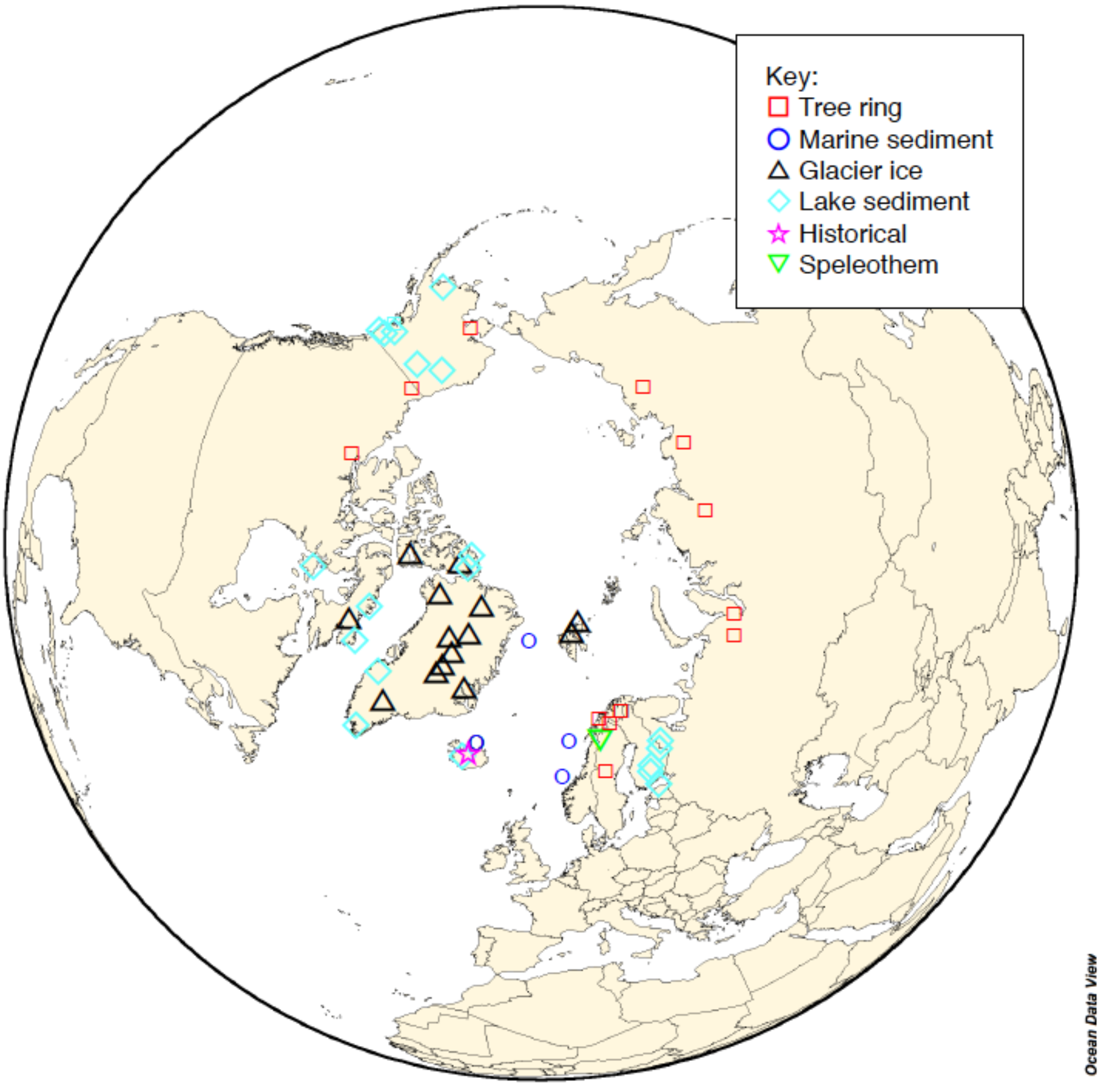

Figure S5. Map showing sites used to reconstruct Arctic annual mean temperatures[39]. Figure S1 was made with Ocean Data View software (Schlitzer, 2015).

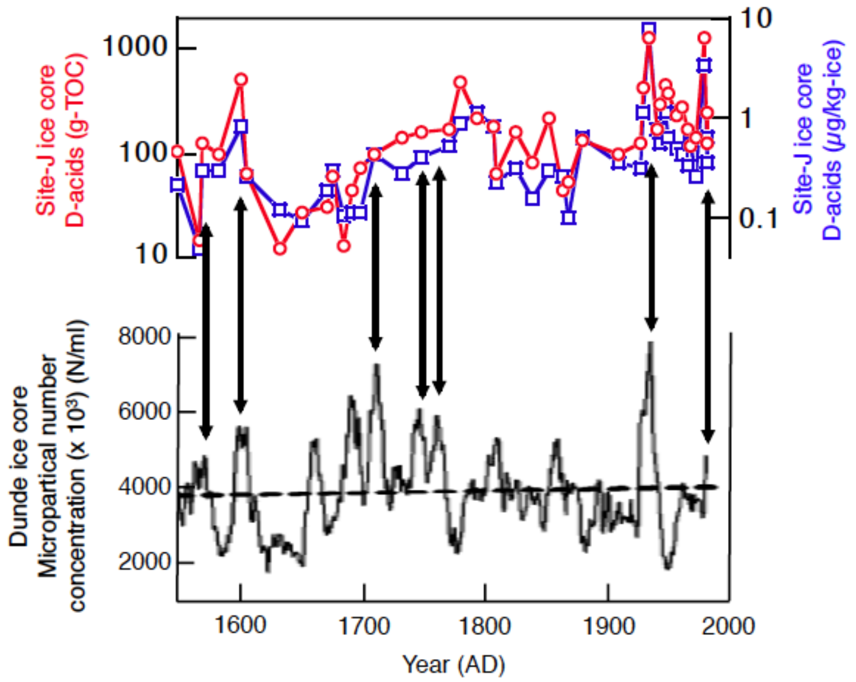

Figure S6. Comparison of Greenland Site-J di-acid records and Dunde ice-core dust record from China (Yang et al., 2006).

References

Acker, J.G. & Leptoukh, G. "Online Analysis Enhances Use of NASA Earth Science Data", *Eos, Trans. AGU* **88**, 14-17 (2007).

Kawamura, K., Suzuki, I., Fujii, Y. & Watanabe, O. Ice core record of fatty acids over the past 450 years in Greenland. *Geophys. Res. Lett.* **23**, 2665-2668 (1996).

Kawamura, K., Kasukabe, H. & Barrie, L. A. Secondary formation of water-soluble organic acids and α-dicarbonyls and their contributions to total carbon and water-soluble organic carbon: Photochemical aging of organic aerosols in the Arctic spring. *J. Geophys. Res.* **115**, doi:10.1029/2010JD014299, 2010.

Schlitzer, R. Ocean Data View, odv.awi.de (2015).

Yang, M., Yaoc, T. & Wang, H. Microparticle content records of the Dunde ice core and dust storms in northwestern China. *J. Asian Mar. Earth Sci.* **27**, 223-229 (2006).
